# Notes on the conservation threats to the western lesser spot-nosed monkey (*Cercopithecus petaurista buettikoferi*) in the Bijagós Archipelago (Guinea-Bissau, West Africa)

**DOI:** 10.1007/s10329-023-01090-9

**Published:** 2023-09-01

**Authors:** I. Colmonero-Costeira, R. M. Sá, M. L. Djaló, N. Cunha, J. Cunha, T. Minhós, I.-R. M. Russo, M. W. Bruford, S. Costa, M. J. Ferreira da Silva

**Affiliations:** 1 Organisms and Environment Division (ONE), School of Biosciences, Cardiff University, Sir Martin Evans Building, Museum Avenue, Cardiff, CF10 3AX UK; 2 InBIO Laboratório Associado, CIBIO (Centro de Investigação em Biodiversidade e Recursos Genéticos), Universidade do Porto, Campus de Vairão, 4485-661 Vairão, Portugal; 3 Program in Genomics, Biodiversity and Land Planning, BIOPOLIS, CIBIO, Campus de Vairão, 4485-661 Vairão, Portugal; 4 Department of Life Sciences, CIAS, University of Coimbra, 3000-456 Coimbra, Portugal; 5 Centre for Public Administration and Public Policies, Institute of Social and Political Sciences, Universidade de Lisboa, Rua Almerindo Lessa, 1300-663 Lisbon, Portugal; 6 Ganogo, Guinea-Bissau; 7 Bissau, Guinea-Bissau; 8 Centre for Research in Anthropology (CRIA-FCSH/NOVA), 1069-061 Lisbon, Portugal; 9 Departamento de Antropologia, Faculdade de Ciências Sociais e Humanas—NOVA FCSH, Avenida de Berna, 26 C, 1069-061 Lisbon, Portugal

**Keywords:** Guenon, Conservation, Habitat conversion, Hunting

## Abstract

The lesser spot-nosed monkey (*Cercopithecus petaurista*) is a widely distributed West African guenon, which is generally considered less vulnerable to local extinctions than many sympatric primate species. Guinea-Bissau harbours the westernmost populations of the species, which is thought to be very rare or even extinct on the mainland, but to have putative populations on some islands of the Bijagós Archipelago. However, due to a lack of regional studies, baseline information on these insular populations is missing. We collected baseline data on the anthropogenic activities that possibly threaten the long-term conservation of this primate by using non-systematic ethnographic methodologies. The species was reported to be decreasing in number or rare by locals on two of the islands, and we identified two main conservation threats to it: generalised habitat loss/degradation, and hunting. While subsistence hunting has been recorded before in these areas, we report, to the best of our knowledge for the first time for these islands, the presence of a semi-organised commercial wild meat trade. The carcasses of western lesser spot-nosed monkeys were observed being stored and shipped from seaports to be sold at urban hubs (Bissau and Bubaque Island). The effect of commercial trade on the species could be severe, considering the small, naturally occurring, carrying capacities typical of insular ecosystems. The results of this study highlight the importance of understanding the leading social drivers of wild meat hunting of lesser spot-nosed monkeys on the Bijagós Archipelago, and the need to conduct baseline research on these insular populations, for which qualitative and quantitative methods could be combined.

## Introduction

According to the International Union for Conservation of Nature Red List of Threatened Species assessment for 2019–2020, more than half of the extant African primate species are under the threat of extinction to some degree. Moreover, most African primate species are experiencing demographic contractions throughout their ranges due to habitat loss and exploitation (Estrada et al. 2017).

The lesser spot-nosed monkey [*Cercopithecus petaurista* (Schreber, 1774)] is the smallest West African guenon (tribe Cercopithecini, family Cercopithecidae). This primate is distributed from Guinea-Bissau and east Senegal to the western part of Togo (Rowe and Myers 2016). Two sub-species have been described—the western lesser spot-nosed monkey (*Cercopithecus petaurista buettikoferi*) and the eastern lesser spot-nosed monkey (*Cercopithecus petaurista petaurista*). Their ranges are separated by the Cavally river, located on the border between the Republic of Liberia and the Republic of Côte d’Ivoire (Rowe and Myers 2016). Until recently, this species was considered less vulnerable to local extinctions than other West African primates due to its socioecological adaptability and small size, which, in theory, should dissuade commercial hunters from targetting it and allow populations to remain at a viable size (Matsuda Goodwin et al. 2020). However, as of 2020, the conservation status of the species was elevated to Near Threatened due to reported increased habitat loss and targeted hunting throughout its range (Matsuda Goodwin et al. 2020). Thus, specific extant populations of this species may be under greater threat than previously thought.

**Fig. 1:**
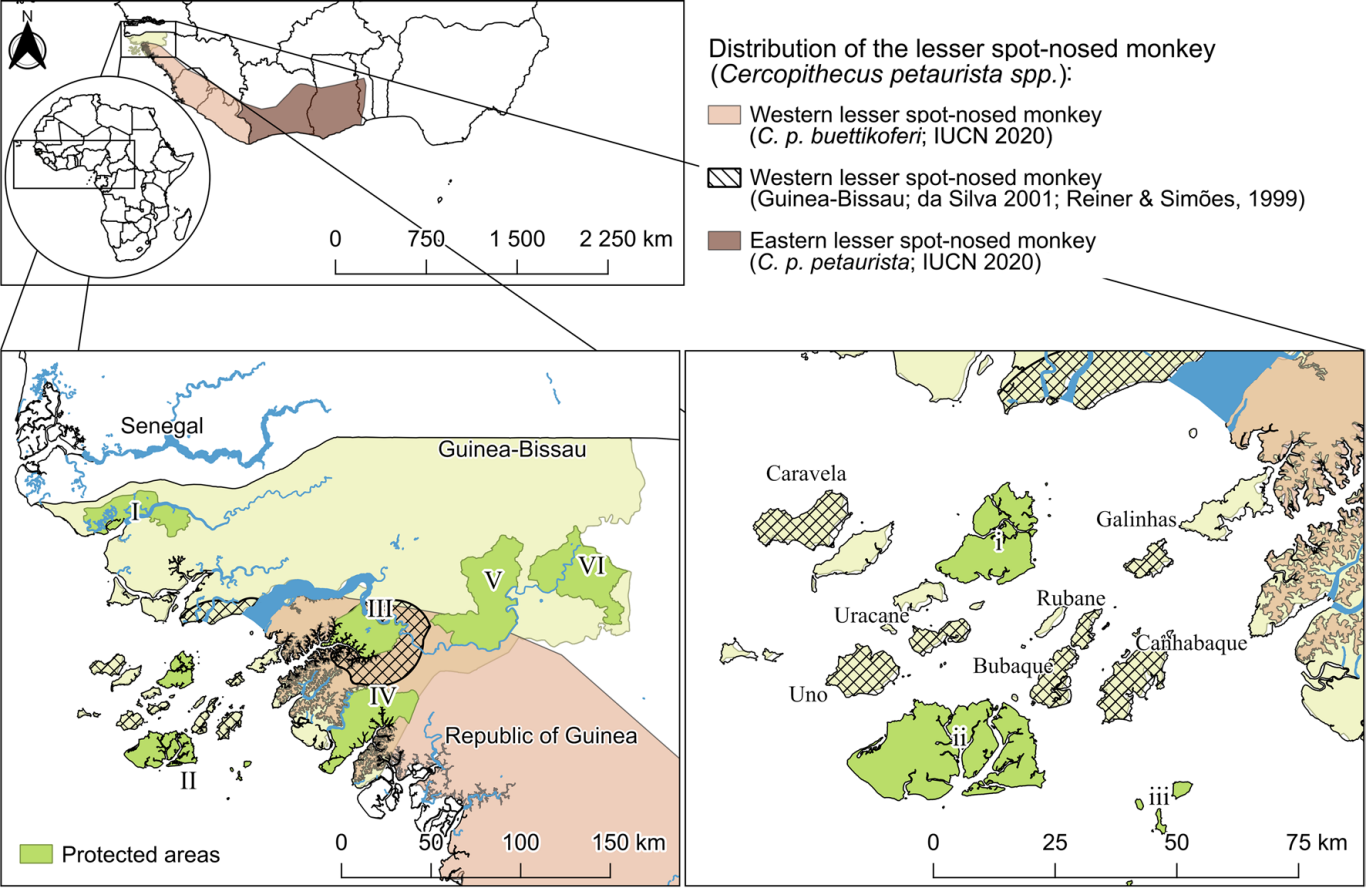
Distribution of the western lesser spot-nosed monkey (*Cercopithecus petaurista buettikoferi*) in Guinea-Bissau. The species distribution is based on polygons constructed by the International Union for Conservation of Nature (accessed in 2023). The distribution of the eastern subspecies (*Cercopithecus petaurista petaurista*) is included. The species distributions are based on regional surveys conducted in Guinea-Bissau (da Silva 2001; Reiner and Simões 1999) and are presented as hatched areas. The maps indicate established protected areas in Guinea-Bissau: Cacheu River Mangroves Natural Park (*I*), Bolama Bijagós Biosphere Reserve (*II*), Cufada Lagoons Natural Park (*III*), Cantanhez Forests National Park (*IV*), Dulombi National Park (*V*), Boe National Park (*VI*), Urok Communitarian Marine Protected Area (*i*), Orango National Park (*ii*), João Vieira and Poilão Marine National Park (*iii*)

Guinea-Bissau is a small West African state (36,125 km^2^) bordered by Senegal to the north and Guinea to the southeast. The country is comprised of the mainland and the Bijagós Archipelago. The western lesser spot-nosed monkey (*santchu bidjugu* or *santchu nariz-branco* in Guinea-Bissau Creole) is likely to be presently restricted to the Bijagós Archipelago (Colmonero-Costeira et al. 2019; Karibuhoye 2004; Gippoliti and Dell’Omo 2003). Previously, the species was present in mainland Guinea-Bissau, Cufada Lagoons Natural Park, and Dulombi National Park (Gippoliti and Dell’Omo 2003; Reiner and Simões 1999) (Fig. 1). At Cufada Lagoons Natural Park, where the species was reported to be hunted for meat consumption (Amador et al. 2015), ex-hunters and guides state that it is currently very rare or possibly even extinct [personal communication (2016), Ferreira da Silva]. At Dulombi National Park, the species has not been detected during recent surveys (Bersacola et al. 2018). The last viable populations of this primate in Guinea-Bissau are thought to live on at most seven of the largest islands of the Bijagós Archipelago (Colmonero-Costeira et al. 2019; Gippoliti and Dell’Omo 2003), none of which are included in currently protected areas (Fig. 1). On these islands, western lesser spot-nosed monkeys are known to live near human communities, and subsistence hunting of the species has been reported (Karibuhoye 2004). Despite the acknowledged rarity of the western lesser spot-nosed monkey in Guinea-Bissau, there have been no conservation actions targeting this species, and baseline information on its populations (e.g. conservation threats, population densities, and genetic diversity) is missing. In this work, we aimed to list and describe anthropogenic activities that possibly threaten the long-term conservation of the western spot-nosed monkey in two of the largest islands where it occurs, based on a non-systematic ethnographic assessment carried out in 2022.

## Methods

### Study area

Our study area included two islands of the Bijagós Archipelago (Fig. 1), which have been anonymised to protect the identity of the local communities and the location of the primate populations. The archipelago covers approximately 10,000 km^2^ and comprises 88 islands and islets of continental origin (Alves 2007). The island closest to mainland Guinea-Bissau is Bolama (located approximately 1.5 km from the coast), and the furthest is Unhocomo (approximately 118 km from the coast). The islands are relatively small, with the largest one covering a total area of 130 km^2^. The climate is similar to that of mainland Guinea-Bissau, and the annual average temperature varies between 25.9 and 27.1 °C. The relative humidity can be as high as 79%, and the average annual rainfall fluctuates between 1500 and 2000 mm. In 1996, the United Nations Educational, Scientific and Cultural Organization recognised the bio-cultural diversity of the archipelago by establishing the Bolama Bijagós Biosphere Reserve (Fig. 1). This reserve currently comprises two national parks (Orango National Park and João Vieira and Poilão Marine National Park) and a protected marine community (Urok Communitarian Marine Protected Area) (Fig. 1).

A quarter of the islands are permanently inhabited by rural communities and another quarter are seasonally cultivated (Madeira 2016). The estimated human population was 32,500 in 2015 (INE 2015). The local communities are dependent on subsistence agriculture of rice and other, secondary, crops [e.g. cowpea (*Vigna unguiculata*) and groundnut (*Arachis hypogaea*)] and cash crops [e.g. cashew (*Anacardium occidentale* L.); Madeira 2016) for their livelihoods.

### Data collection

During the field expeditions, we conducted a non-systematic ethnographic assessment of human activities that potentially involve interaction with the western spot-nosed monkeys. These expeditions were also aimed at collecting faecal samples for non-invasive genetic studies on locally ranging primate groups. During these expeditions, we routinely asked members of the local communities to provide an impression of the demographic trends of the local primate populations and threats to them. Researchers took field notes on their observations when in contact with members of the local communities. When relevant, we took photographic records of the activities if we were authorised to do so by the participants. We conducted a thematic analysis of our notes. This analysis included reading the data thoroughly and reorganising it according to themes as they appeared within the notes (e.g. hunting, perception of primate population size) to gain insights from the observations (Newing et al. 2011).

The methodologies that we used to collect ethnographic data followed the ethical guidelines of the American Anthropological Association. The local communities were informed of our study’s aims and that they could ask us to cease our observations at any stage of our field research. We requested oral informed consent to make and publish our observations before collecting any data. Confidentiality and anonymity were guaranteed to everyone that interacted with the researchers. Original records of field notes were coded for the purposes of anonymity and securely stored. Photographs were only taken after oral informed consent was obtained from the participants. We ensured the anonymity of participants in every photograph taken. The collected data were saved and stored in password-protected electronic documents. Our research received ethical approval from Cardiff University’s School of Biosciences (BIOSI) School Research Ethics committee (SREC) (ref. BIOSI SREC 22-11-01). In Guinea-Bissau, the timeline and the activities and methods used were approved by the Institute for Biodiversity and Protected Areas (Instituto da Biodiversidade e das Áreas Protegidas) and local chieftains. The researchers did not engage in any activity deemed unethical by the International Primatological Society.

## Results

We interacted with the local *Bijagó* communities for approximately 10 days on each island (20 days in total). The length of time spent in the different villages varied between 8 h and 2 days.

On the first island, we visited seven villages. With respect to the perceived demographic trends of the population of western spot-nosed monkeys there, locals consistently reported that these animals were commonly seen, but that the population size was decreasing. From our observations we were able to identify two possible main conservation threats to these populations: habitat loss and/or degradation, and semi-organised hunting.

**Fig. 2:**
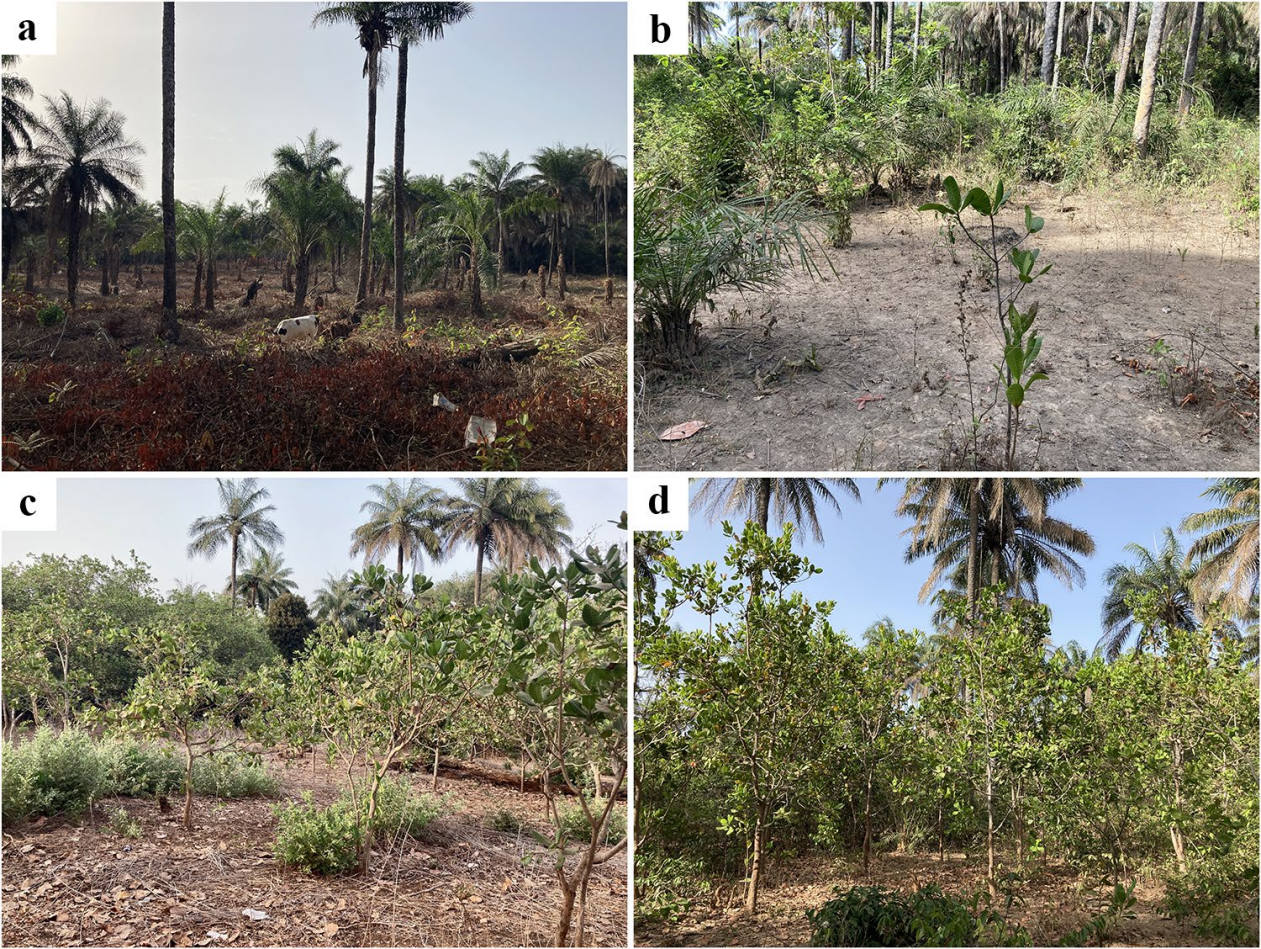
**a**, **b** Habitat conversion on the Bijagós Archipelago. **a** A recently cleared forest patch with grazing cows. **b**–**d** Cashew orchards of increasing age

**Fig. 3:**
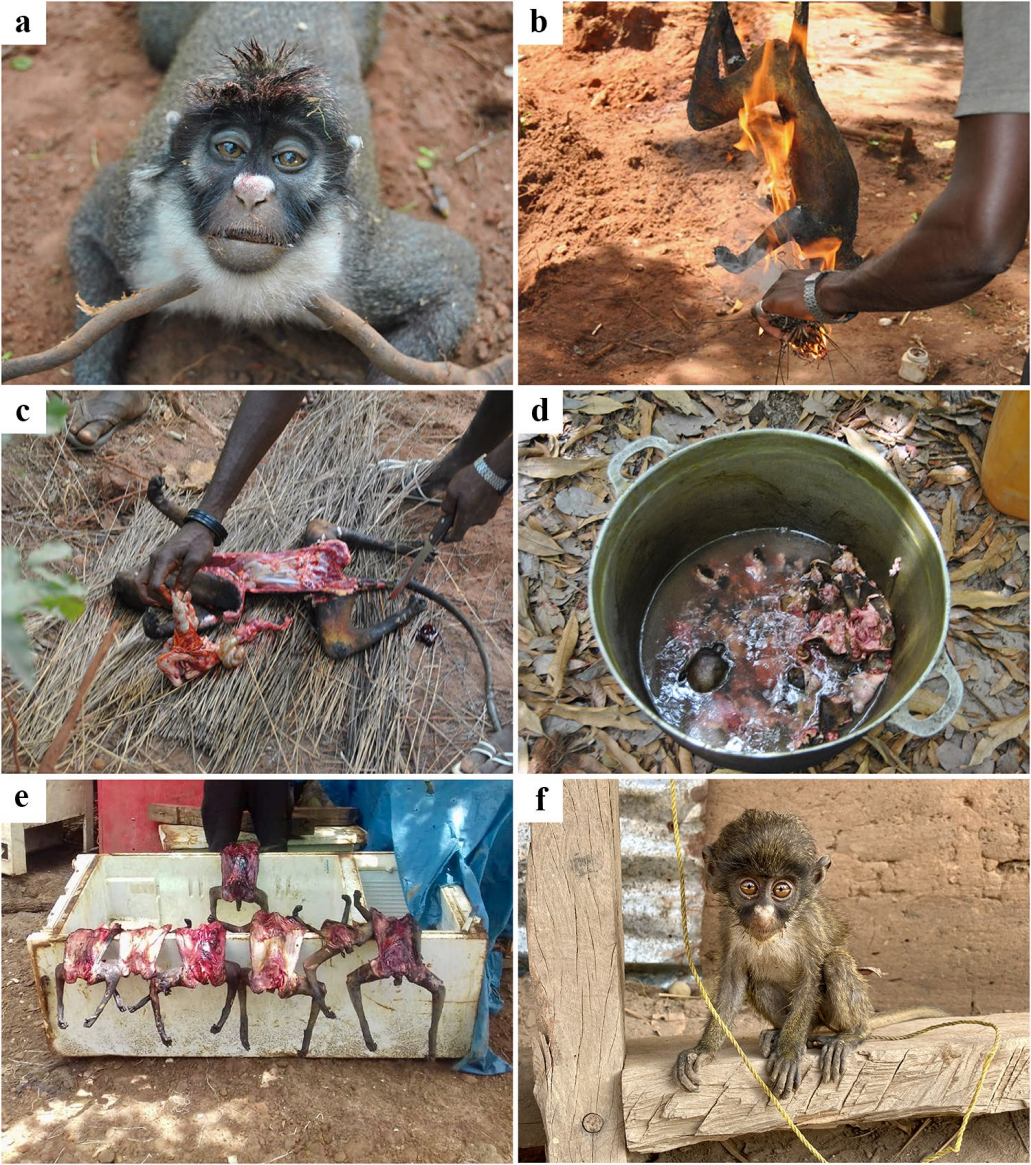
**a**–**f** Wild meat hunting of western lesser spot-nosed monkeys in the Bijagós Archipelago. **a**–**d** Subsistence hunting and preparation of a carcass for consumption. **e** Storage of carcasses for commercial trade. **f** Pet infant monkey caught during a hunting event. (Photos taken by RS and ICC)

Recently cleared patches of forest were common. In addition, there were cashew orchards of various ages in the vicinity of the villages. These extensive monocultures are usually planted in areas previously covered with dry or sub-humid forest patches (Fig. 2). Hunting of western spot-nosed monkeys was conspicuous and widespread. We found the carcasses of recently hunted animals on half of the sampling days in three different villages, on the main road between the villages, and at the main seaport. The number of carcasses found per event varied from one to seven. The hunters were young men (19–35 years of age). The carcasses were smoked and disembowelled shortly after the animals had been killed, and stored whole (Fig. 3a–d). For local consumption, the carcasses were cut up into manageable pieces and added to a stew containing vegetable oil, onion, tomato paste, and chicken stock concentrate, which was cooked for about 1.5 h and served with rice.

The local wild meat trade that was observed was described by locals as a semi-organised commercial practice that included the transportation of carcasses by canoe to the country’s urban hubs (Bubaque Island and Bissau). These boats would arrive from Bissau carrying ice that was deposited in unplugged freezers at the local seaports. The primate carcasses (whole, smoked, and disembowelled) were stored in the ice-filled freezers until transportation (Fig. 3e). Other local terrestrial mammals, namely the Gambian pouched rat (*Cricetomys gambianus*) and Maxwell’s duiker (*Philantomba maxwellii*), were also observed being traded. Although we only observed wild meat trading at the main seaport, testimonies given by the local participants suggested it occurred in at least three seaports on the island. Additionally, we observed a captive infant monkey that had been caught during a hunting event (Fig. 3f).

In the five villages we visited on the second island, locals reported the presence of two primate species, the western spot-nosed monkey and the green monkey (*Chlorocebus sabaeus*). The western spot-nosed monkey was reported to be less common than the green monkey, although some participants mentioned that its population had begun increasing since hunting regulations had been enforced. We observed generalised habitat degradation, with an expansion of cashew orchards in several locations on the island. We did not see direct evidence of wild meat hunting of primates, although locals acknowledged that it does occur. We observed one juvenile and two infant captive green monkeys.

## Discussion

To the best of our knowledge, this is the first study to describe the conservation threats faced by the last remaining populations of the western lesser spot-nosed monkey in Guinea-Bissau due to anthropogenic activities. Previous studies on this species were focused on its occurrence (Colmonero-Costeira et al. 2019; Gippoliti and Dell’Omo 2003) and mitochondrial DNA diversity (Thomas 2011), but a knowledge gap of its local demographic trends, and putative threats to this species, remained.

During our stay on the two islands, we noted several recently planted cashew orchards. This may indicate that there is an expansion of these cash crop monocultures in the archipelago, which replace natural primate habitat. Cashews were introduced into Guinea-Bissau in the 19th century, and now represent the country’s most important agricultural product for export (Catarino et al. 2015; Monteiro et al. 2017). Cashew orchards are typically located in the vicinity of villages. Despite being considered a low-input, low-investment, assured-revenue crop (Temudo and Abrantes 2014), cashew production in Guinea-Bissau is considered unsustainable (Monteiro et al. 2017). Cashew orchards are monocultures, and plant species richness beneath and around the trees is insignificant (Monteiro et al. 2017). Furthermore, due to little agricultural management, cashew orchards are typically characterised by low yields per hectare, possibly leading to an ever-increasing expansion of cultivated areas (Catarino et al. 2015; Monteiro et al. 2017). To the best of our knowledge, no previous studies have addressed the expansion of cashew cultivation in the Bijagós Archipelago. However, our observations suggest that a systematic investigation of the potential impact of this on the country’s insular ecosystems should be conducted.

Subsistence hunting targeting the western spot-nosed monkey had been previously observed in several islands of the archipelago (Ferreira da Silva et al. 2021; Karibuhoye 2004), and commercial trade in these has been mentioned by tourists and inhabitants of the islands (personal observations, RS and MJFS). Here, we report, to the best of our knowledge for the first time, observations of a semi-organised commercial wild meat trade on the archipelago. We also observed a few captive infant/juvenile primates. However, no evidence of trading of captive individuals was observed. Apart from the western chimpanzee, which is explicitly targeted to supply the exotic pet trade, other captive Cercopithecoidea are probably caught during the hunting of lactating females (Ferreira da Silva et al. 2021). Hunting for wild meat consumption in mainland Guinea-Bissau has been interpreted as resulting from low food security and structurally deficient supply chains of fresh meat from farming and fish (Costa 2010). Moreover, a recent increase in wild meat hunting may have been motivated by unstable sources of income, which may encourage young men to engage in this activity, as it has immediate returns (Ferreira da Silva et al. 2021). For example, in some rural areas on the mainland, primate carcasses were reported to be worth between USD 1.27 and 3.69 (Ferreira da Silva et al. 2021), which is a significant amount of money considering that a portion of the country’s population lives on less than USD 2.15 per day (World Bank 2022). Similar motivations may be behind the commercial primate meat trade on the Bijagós Archipelago. Societal dichotomy between younger and older generations has been suggested to be arising within Bijagó communities due to the aspirations of younger generations to abandon traditional sociocultural systems in favour of a more western approach with respect to modernity, personal and regional development (Bordonaro 2006), which potentially favour activities associated with quick economic returns.

The extent to which hunting impacts populations of the western spot-nosed monkey in Guinea-Bissau is currently unknown. Nevertheless, demographic changes related to anthropogenic activities have been reported for other species of primates in the country. Demographic changes reported for other highly hunted species include decreased effective population size (Minhós et al. 2022), increased dispersal distance and preferential gene flow towards areas with a lower population density due to hunting-related mortality coupled with secondary contact between divergent lineages (Ferreira da Silva et al. 2014), and possible disruption of the sex-biased dispersal pattern (Ferreira da Silva et al. 2018; Minhós et al. 2013). However, it is plausible that hunting could impact some populations of the western spot-nosed monkey more severely, as island populations may be intrinsically threatened by genetic factors associated with insularity. Islands are typically colonised by few individuals, which carry only a proportion of the genetic diversity of the source populations (Allendorf et al. 2013). Consequently, insular populations usually display low levels of genetic diversity. Additionally, low or no gene flow between oceanic islands is to be expected. Repeated mating between closely related individuals may result in both increased homozygosity and increased expression of recessive deleterious alleles (Allendorf et al. 2013; Bérénos et al. 2016; Stoffel et al. 2021). A population’s susceptibility to extinction may increase if inbreeding depression (decrease in overall fitness) significantly reduces the population’s viability (Allendorf et al. 2013). Moreover, small islands may not sustain large populations of primates due to their lower productivity compared to larger islands, or continuous ecosystems (Santini et al. 2018). Consequently, the putative commercial trade of this species could prove unsustainable, and should be further investigated. We also highlight that the sustainability and implications of hunting can be better evaluated by conducting comprehensive baseline research on these populations. Specifically, we suggest that assessing the distribution and abundance of the western spot-nosed monkey, as well as estimating its population structure and demographic history based on genetic data, should be priorities.

Our study highlights the fact that non-systematic ethnographic assessments that integrate information from locals as knowledgeable partners can provide baseline data on the conservation threats faced by primate populations. Carrying out relatively fast assessments such as the ones undertaken here can be particularly valuable for understudied, overlooked primate species. However, there is a caveat to the results presented here, as the sampling approach was relatively unsystematic (Puri 2011) and the data were collected over a short period of time, which limits the explanatory power of this study with respect to the described human activities. We stress the need to conduct further qualitative and quantitative ethnobiology research to better understand the main social drivers of the conservation threats faced by these populations of western spot-nosed monkey.

This work could also have further implications for conservation in that it may encourage the development of a local evidence-based conservation action plan specifically for western spot-nosed monkeys, or a concerted action plan for primate species in Guinea-Bissau in general, as many of these are facing similar conservation threats [e.g. Guinea baboon (*Papio papio*) (Ferreira da Silva et al. 2018), western red colobus (*Piliocolobus badius*) (Minhós et al. 2022), king colobus (*Colobus polykomos*) (Minhós et al. 2022)].
